# To facilitate realisation of access, participation, and equity in healthcare: an interview study with policy makers in a Swedish region

**DOI:** 10.1186/s12889-025-23263-5

**Published:** 2025-06-10

**Authors:** Maria Norfjord van Zyl, Margareta Asp, Charlotta Åkerlind

**Affiliations:** 1 Division of Public Health Sciences, School of Health, Care and Social Welfare, Mälardalen University, Västerås, Sweden; 2 Division of Caring Sciences, School of Health, Care and Social Welfare, Mälardalen University, Eskilstuna/Västerås, Sweden

**Keywords:** Access, Equity, Health, Healthcare, Integrated, People-centred health services, Public health, Participation, Qualitative content analysis

## Abstract

**Background:**

How elected healthcare policy makers perceive commonly described core values such as access, participation, and equity, can affect how actions towards these core values will be prioritised. With the example of Sweden, where the healthcare sector in each region is self-governed, this study aims to describe how some Swedish policy makers perceive the prerequisites to facilitate the realisation of access to, participation, and equity in healthcare.

**Methods:**

This qualitative descriptive study involved interviews with ten policy makers, members of a public health and healthcare sub-committee, represented a region in Mid-Sweden. The data collected from the semi-structured individual interviews were subjected to a qualitative content analysis.

**Results:**

The policy makers perceived access as *a service-minded approach*. Considerations about the population’s needs and the effectiveness of healthcare must be addressed to provide access. Participation was perceived as *a reciprocal understanding* where partnership and knowledge were expressed as fundamental aspects of participation. Equity perceived as *a respectful encounter* considers socio-economic preconditions, rights, and continuous endeavour.

**Conclusions:**

The core values are commonly shared values but entails challenges to implement these values in healthcare. Implementation can be facilitated by translating the meanings of the core values into contexts where they are supposed to be applied.

## Background

The right to health is universally acknowledged [1–3] which includes the recurring claims of access, participation, and equality [1] (in this study referred to as equity), as they constitute vital components and core values in health systems [4]. The values can be perceived as existential and fundamental for humans, and central to different health organisations, such as the World Health Organization [5]. Therefore, these core values are commonly used in different types of health-related documents like policies, strategies, and guidelines from organisations such as World Health Organization [4, 5] and European Union [6, 7].

To realize the right to health, public health services and systems as well as healthcare facilities must be effective [8] displaying certain characteristics, such as good quality, equity, and accessibility [4]. These characteristics are in accordance with core values stated by the Council of the European Union [6] and reiterated in the Council Conclusions in 2011 [7]. To provide healthcare to all individuals requires financial arrangements, ambition, and solidarity as well as an ambition to provide healthcare access to all individuals [6, 7] as well as participation [4] and equity [6, 7].

European and global agreements, agendas, and policies can partly be incorporate in Sweden which is a member state of the European Union, the United Nations, and the World Health Organization. Agenda 2030 [9], Health 2020 [10]. In addition, in Sweden, two specific national key documents concerning access, participation, and equity in health care, are the Public Health Bill entitled “Good and equitable health– an advanced public health policy [11], and the Health and Medical Service Act [12].

These are just a few examples of different documents addressing health and core values to strive for. Guidelines for the healthcare sector can be assumed to be based on some of these agreements, agendas and policies.

This study is part of a broader project regarding access to and participation in mammographic screening within a region in Sweden [13–16]. In one of these studies [16] the aim was to describe how policy makers, representing a sub-committee for public health and healthcare, perceive women’s participation in mammographic screening. In the interviews, the policy makers also described their perceptions of the core values on a general level, which is aligned with the focus in the present study.

### The core values access, participation, and equity

A theoretical perspective on the concept of access is described by Penchansky and Thomas [17] as complex and includes different dimensions, such as availability (reflecting services available for the public and their needs), affordability (reflecting costs and fees for the public), accessibility (reflecting distance, cost and means of transportation), accommodation (reflecting waiting hours and contact via telephone) and acceptability (reflecting satisfaction with the provided health service) [17]. Another important dimension for access is, according to Saurman, awareness, which reflects aspects of health literacy and means to improve knowledge [18].

Participation, for instance as the right to participate in decision-making [19–21], is stated in the Council Conclusions and reflects the council’s [6] operational principle of individual involvement. Healthcare with positive health outcomes includes participation with respect of the individual’s rights [22] and autonomy [23].

Equity, according to Whitehead, refers to the absence of avoidable and unnecessary differences with fairness between groups [24]. Regarding equity in healthcare, it is characterised by “fair arrangements that allow equal geographic, economic, and cultural access to available services for all in equal need of care” [8, p.7]. The definition is commonly used, for instance, by the World Health Organization [10, 25, 26] but has been a subject of discussion. For example, in some scoping reviews, the difficulty with the lack of consensus on what health equity is may hinder the realisation of the concept [27, 28].

### Organisations and authorities addressing the core values

The core values of access, participation, and equity, as expressed by the United Nations [1], the World Health Organization [2, 4] and the European Union [6, 7], may be addressed and achieved in different ways for example using a framework such as “ Framework on integrated, people-centred health services as a strategy to realize the core values [29]. Consideration must be taken to each country’s health systems as it may vary in their organisation and set-up.

Sweden’s model of government administration consists of three levels: national, regional, and local. In Sweden, the National Board of Health and Welfare is an example of an authority that assists the Swedish government and healthcare by providing knowledge originating from available research and experiences [30]. The information that the government and the healthcare receive from different agencies, such as The Public Health Agency of Sweden [31], relating to health issues such as unequal access to health care, may result in those issues being brought up on an agenda and communicated to the regions to act upon. The Swedish government has the overall responsibility for healthcare, but the performance of the healthcare delivery and services are delegated to the country’s regions and municipalities [32]. The healthcare system in Sweden has a high universal health coverage. The health care is largely publicly funded and in addition, there is also a yearly cap for out-of -pocket fees for healthcare visits [33]. Each region set the cap for out-of -pocket fees, but in general the fees between the regions are quite similar [34]. In each region, the Regional Assembly is the highest governing body whose members are elected representatives from different Swedish political parties [35], have the responsibilities for health service planning, delivery, funding [36], plans and goals for the organisation [37]. The Regional Executive Committee, appointed by the assembly, leads and coordinates the work within the region and execute the decisions made by the assembly [36]. In this study’s chosen region, committees and sub-committees, appointed by committee, had delegated specific responsibilities [38], such as public health and healthcare. The elected members hold the position for four years corresponding with the term of office.

One responsibility of the sub-committee for public health and healthcare was to monitor the region’s work on national agreements, guidelines and questions concerning quality [39].

They also had the power to make decisions concerning resource allocation up to a certain amount [38].

Studies regarding core values have been mainly addressed separately or in combination. For instance, in comparison to other concepts such as access and equity [40], incorporated in a concept [41], in relation to ethical principals in policy formulation [42], and when commissioning for equity within a health system [43]. According to Kingdon, the agenda setting can be driven by policy makers’ beliefs and ideological factors [44]. The interpretation of the core values for health that are included in policies rests partially on regional healthcare policy makers to adhere to the delegated responsibilities to monitor and follow up on national guidelines, among others. To the best of the authors’ knowledge, no studies regarding healthcare policy makers’ perceptions of prerequisites to facilitate the realisation of access to, participation, and equity in healthcare, has been conducted. The meanings of different concepts may vary between two or more individuals, be affected by sociocultural conditions [45], change over time and sometimes be used as politically correct language [46]. Accordingly, the elected policy makers’ individual perceptions of the core values can have crucial impact on the decisions they make regarding healthcare. The core values may be easy to express verbally or in written form but require more consideration to facilitate the realisation thereof.

Therefore, this study aims to describe how policy makers representing a region in Sweden, perceive the prerequisites to facilitate the realisation of access to, participation, and equity in healthcare.

## Method

A descriptive study using a qualitative approach was conducted, and the data were collected from semi-structured individual interviews [47]. This study as part of a larger research project that focused on access to and participation in mammographic screening [13–15], had a common data collection conducted in 2019 for a paper published in 2021 [16]. A secondary qualitative, inductive content analysis [48] was performed on the collected data for this study. The inductive approach was chosen to analyse the informant’s perceptions of the core values without influencing by theoretical assumptions.

### Setting

The region chosen for the study is situated in Mid-Sweden and has 10 municipalities. Four hospitals are located in four of these municipalities and omennly one hospital offers comprehensive healthcare. The reason for choosing this region was the relevance for the larger research project as the region only had one mammographic screening facility.

### Selection and characteristics of informants

When selecting the informants, a purposeful sampling was chosen [49]. The request for participation in an audiotaped interview was e-mailed to all members of a public health and healthcare sub-committee (n 12) (also holding a position of members in the Regional Assembly) as potential informants. Their assignment was to further investigate certain healthcare issues, with the power to make decisions concerning resource allocation up to a certain amount and to monitor the region’s work on national agreements and guidelines [25]. The email contained an information letter about the purpose of the study and contact details about the first author (MNvZ) if additional information was needed. After 14 days, the invitation was followed up with a telephone call. Six women and four men responded to the request, whereas two individuals did not reply. Reminders were sent to these two individuals one month after the first email. Ten of the 12 members of the public health and healthcare sub-committee were interviewed and had been active as policy makers in the current region for 0.9–19 years. The informants represented six of eight political parties represented in the Swedish parliament. The two unrepresented parties had no elected representative in this specific sub-committee.

The first author (MNvZ) did not personally know the informants prior to the commencement of the study.

### Data collection

An interview guide with pre-set but open-ended questions was used during the individual interviews [50]. Questions about the core values [access, participation, and equity] were discussed in separate parts of the interview but also revisited throughout the whole duration of the interview. The core values were discussed on a general level, but also related to mammographic screening, as example of application. The key questions of the interview were: “How do you perceive the concept of access in healthcare?”, “How do you perceive the concept of participation in healthcare?”, “How do you perceive the concept of equity in healthcare?” and were followed up by probes dependant on the participants answers related to the aim of the study. A pre-test [51] was administered to colleagues to test the questions in the interview guide, and minor modifications such as adding missing words, were made for clarification of the questions. Each informant chose the location of the interview. Eight interviews were conducted in the informants’ respective offices, one over the telephone and one at the regional university. The duration of the interviews was between 37 and 78 min.

The first author (MNvZ) conducted the interviews. Each informant received information about the purpose of the study and the voluntary nature of the participation. All informants also signed an informed consent form in compliance with ethical principles [52]. Short notes were written during each interview to summarise what had been said by the informant. The summary was read to the informant after each interview to ascertain the accuracy of the statements. This facilitated any change, correction, or elaboration of the answers. The interviews were audiotaped and transcribed verbatim by the first author, resulting in a 12–35-page range, in a total of 214 pages. Of these, approximately 130 pages focused specific on the core values. However, all the pages were analyzed to extract the parts that concerned access, participation, and equity.

### Data analysis

The data were analysed, using an inductive content analysis according to Graneheim and Lundman [48]. The first step in the analysis process involved sorting the text into the three content areas– access, participation, and equity– which were then inductively analysed separately. Initially, the transcriptions were read several times to gain an understanding of the data. This was followed by an identification of relevant meaning units, which were condensed. The condensed meaning units were abstracted and labelled into codes according to their contents. The codes were sorted according to their similarities and differences and analysed inductively into sub-categories and categories of each core value (Table 1). A repeated comparison of the data was performed and discussed between two of the authors (MNvZ and CÅ) until consensus was reached.

**Table 1:** Example of analysis

Content area	Meaning unit	Condensed meaning unit	Code	Sub-category	Category
Access	“… from a patient perspective, you should be able to reach healthcare. And that someone is there and responds to my needs at the moment, and depending on how ill I am, that someone is answering on the other side. That one gets a response. That is access for me” (Inf. 10).	You should be able to reach healthcare. That someone responds to my needs. That is access for me.	Response to needs	Need-oriented	Access as a service-minded approach
	“That everyone should have the possibility to get admitted within a reasonable time, to get good care” (Inf. 7).	Care in reasonable time	Reasonable time	Effectiveness	

## Results

This section presents the results of the analysis, derived from the content areas– access, participation, and equity. For clarity, each content area will be addressed, followed by an elaboration of each category and sub-category. The categories are illustrated by quotes from the informants, each of whom is assigned a number in parentheses after each quote. An ellipsis (…) indicates any words or sentences omitted in the quote. Words enclosed in square brackets either clarify the text and verbal expressions or are explanatory notes in cases of pauses or hesitancies by the informant.

Three categories were identified in the analysis: (1) Access as a service-minded approach, (2) Participation as a reciprocal understanding and (3) Equity as a respectful encounter (see Table 2).

**Table 2:** Content areas, sub-categories and categories

Content areas	Sub-categories	Categories
Access	Need-oriented	Access as a service-minded approach
	Effectiveness	
Participation	Partnership	Participation as a reciprocal understanding
	Knowledge	
Equity	Socioeconomic preconditions	Equity as a respectful encounter
	Rights Continuous endeavour	

### Access as a service-minded approach

Regarding the content area *access*, the concept can be perceived as broad, reflecting different interpretations, and expressed as a service-minded approach. Considerations for the needs of the population, as well as the effectiveness of healthcare, have to be addressed to gain access.

#### Need-oriented

The sub-category *need-oriented* covers different aspects, where one basic principle can be considered superior: *“The basic principle of healthcare is that those who have the greatest need for care should receive care first” (Inf. 5).* Additionally, all the policy makers state that geographic distance has an impact on access. For instance, not having access to a vehicle can cause an obstacle as public transportation has to be used. This can include several transfers between bus routes or between different means of transportation, such as by train and bus, before reaching the healthcare facility. If public transportation is needed, some of the policy makers emphasise that it has to cover the geographic areas so as to facilitate access for the members of the population who do not live close to a healthcare facility.

*Of course*,* we shall assure that there is access*,* an infrastructure that works*,* regardless of where you are going. You shall be able to easily travel within the county and also to other counties (Inf. 10).*

However, optimal access also entails closeness to certain types of healthcare facilities, such as primary healthcare. The environment, including the buildings, need to be adapted for social and physical access, or healthcare can be offered in individuals’ own homes. According to all the policy makers, to be responsive to individuals’ needs, access to healthcare can include making contact independent of time by providing solutions (e.g., personal, or digital contact) or making alternative language solutions available.

#### Effectiveness

Receiving high-quality care or treatment within a reasonable time is considered as one aspect of effective access. A reasonable time is determined by the severity of an illness; for instance, certain conditions have a higher priority than others. One obstacle to effectiveness is the backlog of care queues. One reason for backlogs is the lack of competent staff. In turn, one cause of this shortage is the competition for qualified practitioners among different healthcare facilities.

*Eh*,* we try to work the best we can… so that we can employ… people in healthcare*,* but it is difficult… due to competition… from nearby hospitals or municipalities that have university hospitals (Inf. 3).*

Staff is one example of resources; another involves finances. An individual’s financial situation has an impact on effectiveness and therefore access. According to some of the policy makers, effectiveness also necessitates a prediction of demographic changes as population growth causes higher demands on the healthcare system and its resources.

### Participation as a reciprocal Understanding

In the content area *participation*, the policy makers perceive partnership and knowledge as significant aspects and describe the concept as a reciprocal understanding. The individual and the healthcare provider need to be active partners in collaboration, as well as conveyers of information to increase knowledge about different aspects of care.

#### Partnership

The policy makers describe participation as a form of partnership between the individual (patient or relative) and the healthcare professionals. This partnership entails an individual responsibility for self-care, which requires body knowledge, willingness to change certain lifestyle behaviours and being receptive to advice from healthcare professionals. Self-determination is important in the collaboration with healthcare professionals to reach conducive goals, as explained by a policy maker: *“… that you are part of the process between the profession and oneself as an individual*,* that you take part in that process [the healthcare process]” (Inf. 4).*

However, the degree of self-determination depends on the situation, according to some of the policy makers. Depending on the type of disease, the degree of participation varies; for instance, an acute condition may prevent the individual’s participation.

Partnership also includes a shared decision-making. The balance of power between the stakeholders in the partnership has changed over time, from mainly being authoritarian to shared decision-making. Some of the policy makers view the partnership as conditioned by trust; therefore, dialogue is important for the healthcare staff to understand the individual and vice versa. Responsiveness to each other’s experiences and knowledge requires mutual respect and openness, as well as time for questions, for example, considering the local residents’ complaints and viewpoints by acting on these if possible.

#### Knowledge

As stated by all the policy makers, knowledge is a condition for participation and includes continuous, easily understandable, and updated information about the whole procedure for a specific care situation. This can be experienced differently due to various cultural backgrounds. The policy makers explain that information facilitates the utilisation of the healthcare service in an appropriate manner. For instance, the information facilitates an understanding about a medical condition and its treatment, which may increase mental preparedness for what is coming and decrease anxiety. Some of the policy makers also mention that allowing the patient to read one’s own medical records is another source of information. Among groups with low compliance, increased awareness about the necessity and importance of undergoing different health examinations can be achieved by directed educational efforts, according to the policy makers: *“By understanding why you should do it [participate in an examination]*,* you become a participant” (Inf. 6).* Information can be conveyed proactively via an organised event, where the local policy makers meet and talk to the public: *“I believe that information and close contact between people in charge of the healthcare and patients are the most important [for participation]” (Inf. 1).* According to the policy maker, this contributes to the individual’s knowledge and facilitates participation. However, some policy makers mentioned that understanding information requires individual resources, such as physical and cognitive abilities, as well as language skills.

### Equity as a respectful encounter

In the content area *equity*, the policy makers highlight socioeconomic preconditions, rights, and continuous endeavour. These sub-categories display humbleness and acknowledgement for the individual, expressed by a respectful encounter.

#### Socioeconomic preconditions

All the policy makers emphasise that independent of socioeconomic preconditions, the healthcare service should be available for everyone:

*“Yes. For me*,* the concept [equity] is that everyone should receive the care they need without regard to gender*,* sexual orientation*,* religion or skin colour or anything else. Today*,* that is not the case. This does not mean that everyone should have exactly the same [care]; it means that you should receive care based on your own needs*,* and then it will be [equal]… but that is not the case today…” (Inf. 8).*

Social heritage influences the norms, which in turn affect the encounter to be more or less equal between the healthcare service and the individual. Depending on where the individual lives, the healthcare service could be more reachable from the perspective of socioeconomic conditions. This reachability differs not only within regions but also within a city. Gender, functional variations, economics, religion, ethnicity, culture, and education can have impacts on equity:

*“…equal care should be the same for everyone. It also depends on what kind of care it is*,* of course. Acute care should then be more elevated*,* too…. Then it is a human-saving effort*,* so to speak. Then we have a system today*,* where you have the option to sign up for insurance. There*,* you will be first in line*,* for example*,* and so on. It is a system development that has become*,* that if you have more money in your wallet*,* you receive more care than those who do not have money” (Inf. 7).*

According to the policy makers, the lack of ability to retrieve information for the patients and an inadequate understanding of the information are also determinants that can decrease equity in healthcare. Hence, equity can be described as consideration for individual capabilities.

#### Rights

All the policy makers describe that the individual has the right to the best available healthcare, regardless of geographic location, the staff that delivers the care and who you are, which contributes to an overall feeling of safety. Safety benefits not only the well-situated but also the whole society. Additionally, for each individual to have a sense of self-worth, it is important to defend the most vulnerable in society. According to one of the policy makers, this includes the ability to see someone as a person, *ecce homo*, and is not equivalent to equal cases being treated equally. Individuals may assess the lower prioritisation of their healthcare needs as unfair and perceive the situation as a lack of consideration for their individual rights. Furthermore, the policy makers express that individual rights include one’s own responsibility to claim one’s rights, which requires information about what rights one has. One of the policy makers laid more emphasis on the individual’s responsibility than that of the state. In the welfare system, individuals and companies have the option to sign up for additional private healthcare insurance. The policy makers also mention inequity in healthcare among different groups, by giving various examples such as age, diagnoses, social demographics, ethnicity and gender, among others: *“Women in general receive inferior healthcare. The foreign-born do not partake in the same offered healthcare that one may have the right to or need” (Inf. 8).* The gender perspective is addressed by some of the policy makers, for instance that women in general do not prioritise themselves, in favour of the family and the employer. The women’s rights are expressed as perhaps not being fully utilised, which is even more apparent among women who have less education and are older and blue-collar workers. It is mentioned that different perspectives can be applied when addressing equity in healthcare, and the healthcare authorities should be observant, focusing on groups that already face difficulties: *“… the surrounding society has to [provide] support to get everyone to join*,* so that it becomes equal” (Inf. 9).* The responsibility weighs heavier on society than the individual when it comes to advocacy of rights for different groups.

#### Continuous endeavour

Equity as a concept has intrinsic difficulties as it is challenging to measure and perceived as a “soft value”:

*“… right now*,* one has to invest in very hard values and this [equal care] is a very soft value…hard values*,* I think*,* is more buildings*,* real estate*,* things like that…” (Inf. 2).*

Most of the policy makers also affirmed that equity in healthcare is a continuous endeavour that encompasses long-term planning, more gender-equal political representation, ongoing discussions, and active engagement in obtaining follow-up data concerning different equity indicators. Clear goals are other means to attain equity in healthcare and are included in regional plans: *“We make decisions and work through them [policies concerning equity in healthcare]” (Inf. 10).* However, some of the policy makers point out the lack of information on how the staff adhere to the set goals in the regional plans. The endeavour to achieve equity in healthcare includes prioritising. Sometimes, it requires political courage to deviate from adopted policies to do something different. A fair allocation of resources is also important when addressing equity as it is funded with taxes. The public’s increased expectations about healthcare due to technical and medical advancements, in combination with limited resources, are acknowledged by the policy makers as putting a strain on achieving equity in healthcare. The ambition to achieve equal and timely healthcare causes a conflict between reality and vision due to the lack of resources.

## Discussion

This study aims to describe how policy makers, representing a region in Sweden, perceive the prerequisites to facilitate the realisation of access to, participation, and equity in healthcare. These concepts and core values stated by different organisations [1–3, 5–7] and based on the understanding by the policy makers are reflected in three categories: access as a service-minded approach, participation as a reciprocal understanding, and equity as a respectful encounter.

The policy makers perceive the three concepts of access, participation, and equity as complex and to a certain extent, difficult to define and measure. It is assumed that this can become a problem if comparisons are needed among regions at both the national and international levels. The lack of consensus on the definitions of the core concepts may also cause difficulties in operationalising them. To a certain extent, this argument is supported by the World Health Organization [53] which states that core concepts need to be comparable and measurable [54]. Also, Wenzl [43] support the need for a consistent definition because the lack thereof may jeopardise the implementation of the concept. Another threat may be the policy makers and decision makers beliefs on what is worth prioritising. Prioritisation can be linked to values; for instance, Schwartz [55] asserts that the values that are important to strive for motivate actions and can be understood as worth prioritising.

The categories *access as a service-minded approach*, *participation as a reciprocal understanding*,* and equity as a respectful encounter* can be implemented for instance by using an integrated, people-centred health service [29, 56], considering both the individual and the population perspectives.

### Perspectives on access

This study’s results show that *access* as a concept requires a service-minded approach since it caters to the needs of individuals and aims for the effectiveness of the healthcare system. For instance, fulfilling the needs of individuals requires geographic and physical access, as well as a flexible service, and the individual circumstances are considered. To apply a service-minded approach, a reorientation of the health services towards a more integrated people-centred health service [29] can be a solution. It addresses, not only what is facilitated within the healthcare facility, but the dimensions *accessibility*, *availability*, and *accommodation* of the concept access [22] as a social determinant that affect the health of the population outside the healthcare. Understandably, the facilitation of service-minded approach lies further away from the individual’s own control as it requires more financial resources and political will. This is a task for policy makers at the national, as well as the regional and local level.

Effectiveness, in the current study’s result, concerns essential resources, such as staff and time; for instance, a prolonged time before receiving diagnosis and treatment may aggravate the patient’s condition and reflects accessibility, which considers, among others, travel time and distance [17].

Everyone cannot live close to a healthcare facility, with a risk of not having timely access to healthcare, related to their needs. Hence, a conflict with the core value of access for all individuals [6, 7] may arise. This reflects the theoretical perspective on the concept of access and the availability dimension identified by Penchansky and Thomas [17].

### Perspectives on participation

A partnership based on a *reciprocal understanding* is presumably valuable in the endeavour for participation regardless of whether it is the patient– healthcare provider relationship, or the population in relation to the healthcare system. This partnership may facilitate health decisions, however informed decisions require knowledge, which can be conveyed via education, information, and other sources [57], and can facilitate participation in decision-making.

Trust in different sources of information is also found to be related to the level of health literacy, where individuals with lower health literacy tend to prefer different social media over medical sources [58]. This could be a cause for concern as poorly informed health decisions may influence participation and consequently, the health outcomes. In the current study, participation can be regarded from the perspective of decision-making about individual treatment, and as an individual responsibility for self-care. Another perspective is expressed by Aslani [59], where participation is needed to engage the individual in decision-making regarding one’s own health. Common for these different perspectives is that all require knowledge and health literacy to perform conducive self-care or make well-informed decisions. Health literacy [60] and partnership for co-production of health are for instance addressed in integrated, people-centred health services [29].

### Perspectives on equity

In this study equity is translated into respectful encounter, which includes socioeconomic preconditions, rights, and a continuous endeavour. The preconditions, sometimes referred to as social determinants of health [61] are pivotal for equity, and in this study, education, income, social heritage, residential area, and gender are addressed. Among others, these have also been identified in some studies and reports as central when addressing equity in health [8, 62, 63]. The social determinants affect the realisation of the right to health. Marmot stresses, “Social determinants of health and access to healthcare are separable, but not separate issues in practice” [61^(p 195)^], which in this study can be seen as reflecting an integrated people-centred health services and could be assumed to implicitly be part of a continuous endeavour for health. To prevent health problems, such as in a population, there is a need to understand the determinants of health [64] and a health service such as primary care, where the relation between public and personal health is established since services are offered both at an individual level and from a public health perspective [65].

In this study, the individual’s right to the best available care is aligned with the core value of access to healthcare of good quality [6, 7]. Additionally, the individual’s autonomy, sometimes also reflecting one’s own responsibility, and can presumably be linked to the right to decide [19–21]. This in turn can be an expression of respect for fellow human beings [23]. However, it is important to understand that not all groups possess the capabilities to exercise their rights, and according to Solar and Irwin [66], society has the responsibility to advocate the rights of different groups. As the results from the current study shows, society has the responsibility to step up to the plate to advocate the rights of different groups when the individual cannot do so. In this case, the society is those in power to make decision further beyond the individual’s reach, such as policy makers. Even though current study did not aim to compare the perceptions of the concepts in the light of politics, but from the informant’s personal view, the personal values influence the political beliefs [67] hence decisions. Values are based on beliefs and are important to motivate actions that can be understood as worth prioritising [55]. This is supported by Kingdon’s [44] statement that policy makers’ beliefs and ideological factors may influence the agenda setting. Therefore, it is supposed that a consensus among policy makers to protect the rights of different groups is important in the endeavour to achieve equity in health.

The policy makers mention that the endeavour for equity requires long-term planning and resource allocation. Additionally, active engagement is needed when following up on the data concerning equity. This could be assumed as contributing to the knowledge base about the social determinants of health, referring to all the social circumstances in which people live their everyday lives [66]. Both these actions correspond to the recommendations of the Commission on Social Determinants of Health [68] and can be perceived as having positive impacts on the effort to increase health equity and consequently, to actively work for adherence to the core value of equal rights [53].

This study’s result reflects the complexity of the concepts of access, participation and equity. They can be described separately; however, they are often entwined. For example, for something to be equal, it requires access in all its dimensions, as well as participation. Participation, which encompasses autonomy and the right to make well-informed decisions, requires access to information and the ability to understand and act on the information given, which reflects health literacy. All of the above require equal access and participation in reality, not only as written ambitions. In general, the perceptions among the policy makers regarding prerequisites to facilitate the realisation of access to, participation, and equity in healthcare, were similar. This could be due to the universality of the core values.

### Implications

Based on the results of our analysis and with support of the background an understanding of the core values access, participation, and equity, may be facilitated by applying a service-minded approach, reciprocal understanding, and a respectful encounter, allowing the core values to be more practically manageable. In order to achieve this, for instance formulating policy documents regarding these core values may facilitate the operationalisation thereof. In the process of developing the policy documents it is of importance to include different stakeholders relevant for the context in healthcare and public health services. This could be in the form of workshops, seminars, and citizen dialogues. This in turn, implies that regional network building [69], can be one possible solution to facilitate the diffusions of the core values’ meaning at different organisational levels, from a top-down and bottom-up approach.

To reduce health inequities, a broader perspective on the social determinants of health should be applied [70] by healthcare policy makers, on a regional, national, and international level, as the determinants comprise both access and participation [7]. A regional perspective is needed as the conditions for resource allocation and demographics differs. This broader perspective is important to bear in mind as Sen [71] emphasises that health determinants extend further than the scope of healthcare. The determinants could be monitored by using relevant regional statistic data, as well as distribution of surveys regarding demographic conditions.

### Strengths and limitations of the study

Trustworthiness is of importance in qualitative studies why credibility and transferability must be discussed [72]. To achieve credibility, two of the researchers participated in the analysis process and discussion until consensus was reached. The results highlight the complexity of the values in focus, and it is for the reader to assess if the result can be transferred to similar settings, understood as credibility [48].

To achieve credibility, informants need to have experiences of the phenomenon under study [73]. This can be viewed as a strength of the study because the informants have broad knowledge of the demographics of the region, which may have impacts on access, participation, and equity, which constitute the phenomenon under study. The informants are responsible for individuals’ healthcare, with different geographical conditions to consider when decisions must be made. Another strength is that 10 of the 12 members of the public health and healthcare sub-committee chose to participate in the study, and there was an even mix of gender and the number of working years.

To achieve dependability [73] an interview guide with pre-set open-ended questions was used so that all participants were asked the same questions, while having the opportunity to answer them freely based on their individual experiences.

Since two researchers were involved in the analysis process, their interpretations varied, and their pre-understandings were revealed during the discussions. To facilitate the reader’s ability to judge the credibility of the study, examples of the interpretation processes have been presented [73].

The meanings of access as a service-minded approach, participation as a reciprocal understanding, and equity as a respectful encounter can be useful. However, the implementation of their meaning can vary, depending on the prerequisites in the society and determinants for health.

The meanings of the core values are intangible of nature and perceived as interdependent which posed challenges during the analysis process. This may have influenced the understanding of the core values which can be regarded as a limitation of the credibility.

The healthcare system in Sweden is decentralised to some extent, similar to other European countries, such as Belgium, Croatia and Finland. In these countries, are some of the responsibilities delegated from the national government to communities/municipalities and regions [74]. However, it can be assumed that the core values are of significance regardless of healthcare governance and therefore facilitate transferability of this study’s result to other countries.

In this study the informants’ personal perceptions of the concepts were in focus, not their political affiliations. Even though the interviews were “fairly” long, the policy makers personal understanding could have been difficult to reach as they most likely are used to be interviewed in their role as policy makers. Therefore, the separation between personal opinions and political correctness [46] might be difficult to achieve and could potentially be perceived as a limitation. However, the policy makers never expressed themselves as political spokespersons by using statements such as “we in our party…”, “we stand for…”.

## Conclusions

The core values of access, participation, and equity may be facilitated for by adopting a service-minded approach, reciprocal understanding, and respectful encounter respectively.

Even though the core values are shared values, there are also shared challenges to implement the values in a context. A conclusion is that the implementation of the core values may be facilitated by translating them into the context where they are supposed to be applied.

In addition, it can be of value for Sweden and other countries to work towards a reorientation of the health system, for instance an integrated, people-centred health services may facilitate a realisation of the core concepts as it addresses all of these.

## Data Availability

The reason behind the decision to not make the data publicly available is the protection of the informants’ confidentiality. For any information regarding the transcripts of the interviews or the data analysis, the corresponding author may be of assistance.
